# A Real-Time Method for Improving Stability of Monolithic Quartz Crystal Microbalance Operating under Harsh Environmental Conditions

**DOI:** 10.3390/s21124166

**Published:** 2021-06-17

**Authors:** Román Fernández, María Calero, Yolanda Jiménez, Antonio Arnau

**Affiliations:** 1 Advanced Wave Sensors S.L. Paterna, 46988 Valencia, Spain; 2 Centro de Investigación e Innovación en Bioingeniería, Universitat Politècnica de València, 46022 Valencia, Spain; macaal3@teleco.upv.es (M.C.); yojiji@eln.upv.es (Y.J.); aarnau@eln.upv.es (A.A.)

**Keywords:** monolithic quartz crystal microbalance, biosensor, discrete wavelet transform

## Abstract

Monolithic quartz crystal microbalance (MQCM) has recently emerged as a very promising technology suitable for biosensing applications. These devices consist of an array of miniaturized QCM sensors integrated within the same quartz substrate capable of detecting multiple target analytes simultaneously. Their relevant benefits include high throughput, low cost per sensor unit, low sample/reagent consumption and fast sensing response. Despite the great potential of MQCM, unwanted environmental factors (e.g., temperature, humidity, vibrations, or pressure) and perturbations intrinsic to the sensor setup (e.g., mechanical stress exerted by the measurement cell or electronic noise of the characterization system) can affect sensor stability, masking the signal of interest and degrading the limit of detection (LoD). Here, we present a method based on the discrete wavelet transform (DWT) to improve the stability of the resonance frequency and dissipation signals in real time. The method takes advantage of the similarity among the noise patterns of the resonators integrated in an MQCM device to mitigate disturbing factors that impact on sensor response. Performance of the method is validated by studying the adsorption of proteins (neutravidin and biotinylated albumin) under external controlled factors (temperature and pressure/flow rate) that simulate unwanted disturbances.

## 1. Introduction

Conventional analytical methods currently employed as a “gold standard” require trained personnel in centralized laboratories to perform time-consuming experiments with costly, large, and bulky devices. Owing to their simplicity, reduced size, good sensitivity and low cost, novel biosensors may play a fundamental role in the very near future, becoming an alternative analytical tool in health care, food security and environmental monitoring applications.

Biosensors can be classified by their transduction mechanism. Nirsch et al. provided a comprehensive overview about sensor transducer principles for label-free biomolecular interaction analysis [1]. Although novel transducers are continuously emerging, electrochemical, optical and acoustic transducers are the most popular ones. These three approaches are well-established technologies with their advantages and drawbacks (see references [1,2] for more information). 

Among acoustic biosensor technologies, quartz crystal microbalance (QCM) stands out as a direct label-free detection tool suitable for real-time monitoring. QCM operation is based on the so-called gravimetric technique [3], which relates mass changes on the sensor surface to resonance frequency shifts, Δ*f*_r_. This approach has been widely applied in bio-chemical sensing: immunoassays, protein adsorption, DNA hybridization, etc. [4,5,6,7]. If the dissipation parameter, Δ*D*, is also monitored (QCMD), viscoelastic and conformational properties of the sample can be studied as well [8].

Recently, an enhancement in the limit of detection (LoD) of around two orders of magnitude has been reported for high fundamental frequency QCMD (HFF-QCMD) sensors with resonance frequencies of up to 150 MHz [9,10,11,12,13,14]. The HFF-QCMD principle of operation relies on the reduction of the quartz plate thickness of a classical QCMD [14], resulting in a sensitivity increase and a surface reduction [15]. Thanks to their small footprint, it is possible to integrate dozens of HFF-QCMD sensors within the same substrate through the design of Monolithic QCM arrays (MQCM) [16]. Miniaturized and parallelized elements in the array lead to relevant benefits including high throughput, lower cost per sensor unit, less sample/reagent consumption and faster sensing response [17,18,19].

Although MQCM technology is well-suited for biosensing, there are still remaining challenges that hamper its adoption for portable applications. Some effects intrinsic to the sensor setup (such as mechanical stress exerted by the measurement cell or electronic noise of the characterization system) and external factors (such as temperature, humidity, vibrations, or pressure) can strongly affect sensor stability [20], masking the signal of interest and degrading LoD. Isolating the sensor response from those factors is not trivial and increases the complexity and cost of the testing equipment, often preventing the development of lightweight and portable instruments appropriate for real-time applications. 

Changes in pressure can be a consequence of the pumping system (i.e., typical “peaks” with syringe-pumped fluidic systems [15]), or of a change in flow rate of the fluidic system, i.e., when regenerating the sensor surface in an inmunoassay [9,12]. Changes in room temperature also have a significant influence on the HFF-QCMD resonators response. In the case of temperature regulation, active thermal control systems, usually based on Peltier thermoelectric modules, are common. There have been attempts to find alternative solutions to the use of bulky, expensive and complex control systems. Mecea et al. proposed the use of two QCM resonators simultaneously, one of which is used as a reference [21]. The effect of temperature fluctuations in the resonance frequency is cancelled by directly subtracting the signals from both sensors. The Mecea method significantly reduces temperature disturbances when two conditions are met: (1) the temperature is equal at both resonators, and (2) the response of both resonators versus temperature matches. Rahtu et al. [22] compared the aforementioned method with a numerical correction of the temperature effect based on a third-degree polynomial model. Rahtu’s method provided similar performance without the necessity of a reference QCM resonator. However, it only proved to be valid for monotonous temperature variations and showed convergence issues. Furthermore, this scheme requires an additional temperature sensor. Pierce designed an insensitive quartz microbalance based on stress-compensated (SC) crystals [23]. By simultaneously measuring the resonance frequency of two overtones at a single resonator and assuming a gravimetric regime, it was possible to calibrate the crystal response to temperature without the need for an external thermometer. The main disadvantage of SC-cut resonators is their higher cost due to the complex double rotation process required for their manufacturing.

In a real portable biosensing device, not only the temperature but also many other factors can concurrently undermine the sensor stability, leading to a lack of reliability in the measurements. In this case, a more complex signal conditioning stage, combining different strategies, is required. At some point, selective filtering can be applied [24]. It tends to work well to reduce high-frequency fluctuations (noise) but fails in removing low-frequency disturbances (drift), whose spectrum often overlaps with the signal components of interest. Advanced statistical methods, such as principal component analysis (PCA), can also be used. PCA is based on the dimensional reduction of a large dataset to highlight its most statistically significant components, which ideally are related to the signals of interest, while removing the less significant components, which could be associated with frequency instability. Lately, Corradi et al. applied the PCA method to improve the detection limit of a QCM sensor operating in multiple overtones [25]. Mumyakmaz et al. combined PCA with neural networks to compensate for the effect of humidity in toluene gas monitoring [26]. However, due to their relatively high computational cost, PCA-based approaches are not generally used for signal correction in real time, but for pattern recognition purposes in post-processing stages.

It is important to mention at this point that although dissipation is a very valuable QCM parameter that provides information about viscoelastic and conformational characteristics of the sample, all methods we found in the literature are focused exclusively on improving the quality of the resonance frequency.

Here, we present a method based on the discrete wavelet transform (DWT) to improve the stability and reliability of resonance frequency and dissipation signals of QCMD sensors. DWT is the discretely sampled version of the continuous wavelet transform. Wavelets are mathematical functions widely used for time-frequency analysis of transient, non-stationary and time-varying phenomena [27]. Wavelet analysis is based, as with Fourier theory, on the concept of signal approximation using superposition. A general way of looking at wavelet functions is as families of functions with an excellent resolution in both frequency and time. This feature makes wavelet analysis a powerful tool in signal processing applications. The algorithm introduced in this paper takes advantage of the high similarity found among the noise patterns of the resonators integrated in an MQCM device to mitigate both intrinsic and environment factors that impact on the sensor response, thus effectively improving LoD in portable applications. This approach is suitable for real-time signal correction because of its low computational cost.

To validate the performance of the method, we first investigated its capability to reduce frequency instability caused by sensor intrinsic factors. For that, we simulated a basal state of the setup by making a system to work under controlled and static external conditions of temperature and pressure. Then, we extended our investigation to evaluate the method under a harsh environment, simulated by high temperature and flow rate gradients. Under these conditions, we monitored a protein adsorption experiment. Label-free detection of protein–ligand interactions is one of the paramount applications of QCM [28]. In particular, we selected neutravidin (NAv) and biotinylated bovine serum albumin (bBSA) as a relevant model system since these proteins are commonly used in biotechnology and bioanalytics to prepare the sensor surface for further chemical modification [29,30,31,32,33].

## 2. Principles of the Method

Generally, DWT is calculated through the so-called Mallat tree decomposition scheme [27], which is based on a quadrature mirror filter that splits the signal into two sub-bands. After a decimation by 2, the high-frequency sub-band is termed as the detail coefficients and the low-frequency sub-band as the approximation coefficients. This process can be repeated sequentially, generating several decomposition levels (see Section S1 of the Supporting Information). DWT is a reversible process and after the transformation, the original signal can be recovered from the whole set of detail and approximation coefficients.

**Figure 1:**
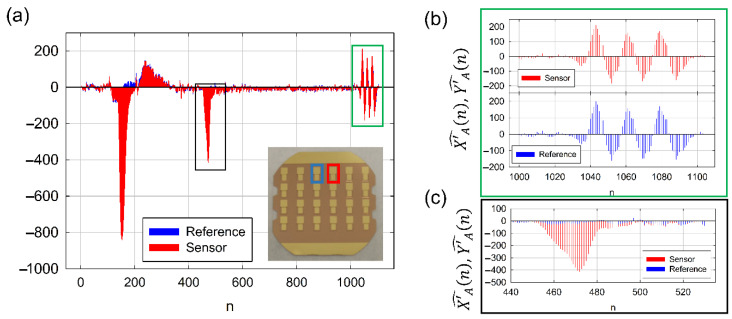
(**a**) Time derivative of the frequency shift approximation coefficients for the sensor (red) and the reference (blue); n refers to the sample index (**b**). Detailed view of the green inset in figure (**a**). (**c**) Detailed view of the black inset in figure (**a**).

When DWT is applied to frequency and dissipation signals measured at a pair of resonators integrated in an MQCM device, an interesting effect is observed in the approximation coefficient series that it is not clearly apparent from raw data. A high linear correlation level is found in the time derivative of the approximation coefficient between neighbor resonators if they are subjected to similar environmental conditions (see green inset in Figure 1a and its detailed view in Figure 1b). In contrast, when a sample is injected only through the *sensor* but not through the *reference* (black inset in Figure 1a), time derivatives differ significantly, as can be seen in the detailed view of Figure 1c. It is important to note that although the sensors are very close to each other (<2 mm) and they are integrated in the same quartz substrate, their absolute response to those external factors differ to some extent. Based on the above observations, we assume that linearly correlated variations in DWT time derivatives of neighbor resonators are caused by unwanted external effects. Hence, we propose a method that combines DWT analysis with an algorithm that extracts and cancels out these correlated factors to improve both resonance frequency and dissipation stability.

## 3. Materials and Methods

### 3.1. Description of the Method

To introduce the method, let us consider two resonators as the ones depicted in Figure 1a. One of the resonators, which we call *sensor*, is exposed to the sample, while the other, which we call *reference*, is kept isolated from it. We call *x*(*n*) the sampled signal in time domain (either resonance frequency or dissipation) measured at the *sensor* and *y*(*n*) the same sampled signal measured at the *reference* resonator. The method provides a corrected output signal called *z*(*n*). *n* refers to the sample index.

**Figure 2:**
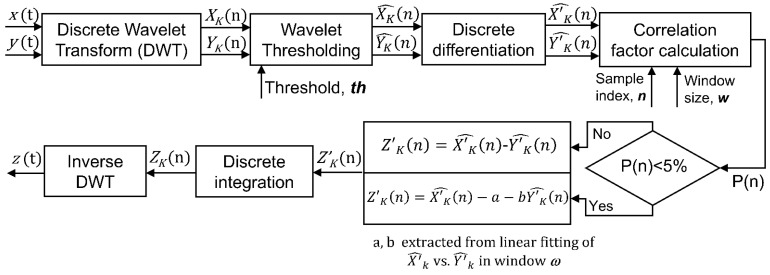
Flow diagram describing the different steps of the method.

Practical implementation is depicted schematically in Figure 2 and described next:

**STEP 1:** We calculate the DWT of *x*(*n*) and *y*(*n*). Without loss of generality, we apply the Daubechies wavelet transform with three vanishing moments (db3) and four decomposition levels [34]. This transform will generate five series of coefficients: we use $X_{K}\left(n\right)$ and $Y_{K}\left(n\right)$ to refer to the whole set of wavelet coefficients of the *sensor* and the *reference* respectively; *k* = A, D_1_, D_2_, …, D_q_. (see Equation (1)).

(1) $$\begin{array}{c} x\left(n\right)\overset{DWT}{\to }X_{A}\left(n\right),X_{D1}\left(n\right),\;X_{D2}\left(n\right),X_{D3}\left(n\right),X_{D4}\left(n\right)\\ y\left(n\right)\overset{DWT}{\to }Y_{A}\left(n\right),Y_{D1}\left(n\right),\;Y_{D2}\left(n\right),Y_{D3}\left(n\right),Y_{D4}\left(n\right) \end{array}$$

where $X_{A}\left(n\right)$ and $Y_{A}\left(n\right)$ refer to the approximation coefficients expansion; $X_{Dq}\left(n\right)$ and $Y_{Dq}\left(n\right)$ represent the detail coefficients expansions of the different decomposition levels q (q = 1 to 4) (see Figure S1 of the Supporting Information). Each one of these coefficient series captures the behavior of the signal in a certain frequency sub-band.

**STEP 2:** We use the so-called wavelet shrinkage technique [27] to eliminate all those detail wavelet coefficients that do not make a significant contribution to the total energy of the signal (see Supporting Information Section S2). To that end, hard-thresholding is applied [27]. The threshold components are named ${\hat{X}}_{K}\left(n\right)$ for the *sensor* and ${\hat{Y}}_{K}\left(n\right)$ for the *reference*. 

**STEP 3:** Numerical time derivatives of the threshold components are calculated for the *sensor* (${\hat{X^{\prime }}}_{K}\left(n\right)$) and the *reference* (${\hat{Y^{\prime }}}_{K}\left(n\right)$).

**STEP 4:** Pearson product-moment correlation coefficient, *R,* is calculated between ${\hat{X^{\prime }}}_{K}\left(n\right)$ and ${\hat{Y^{\prime }}}_{K}\left(n\right)$. A window of *w* samples around each sample *n* is considered for the calculation. The correlation coefficient is determined by dividing the covariance of ${\hat{X^{\prime }}}_{K}\left(n\right)$ and ${\hat{Y^{\prime }}}_{K}\left(n\right)$ by the product of the two variables’ standard deviations according to Equation (2).

(2) $$R\left(n\right)=\frac{Cov\left({\hat{X^{\prime }}}_{K}\left(n\right),{\hat{Y^{\prime }}}_{K}\left(n\right)\right)}{\sigma_{{\hat{X^{\prime }}}_{K}}\sigma_{{\hat{Y^{\prime }}}_{K}}}$$

Then, the *p*-value *P*(*n*) is obtained from *R* to determine whether or not the correlation between the *sensor* and *reference* is statistically significant. The *p*-value is computed by transforming the correlation to create a t statistic having *w*-2 degrees of freedom. The confidence bounds are based on an asymptotic normal distribution of 0.5 × log((1 + R)/(1 − R)).

**STEP 5:** We use ${\hat{X^{\prime }}}_{K}\left(n\right)$ and ${\hat{Y^{\prime }}}_{K}\left(n\right)$ to calculate the derivatives of the wavelet coefficients of the new corrected signal ${Z^{\prime }}_{K}\left(n\right)$. If the ${\hat{X^{\prime }}}_{K}\left(n\right)$ and ${\hat{Y^{\prime }}}_{K}\left(n\right)$ components are statistically related in a significant way (*P*(*n*) < 5%), the derivative of the corrected coefficient is calculated as a linear projection of the reference space into the sensor space:

(3) $${Z^{\prime }}_{K}\left(n\right)={\hat{X}}^{\prime }{}_{K}\left(n\right)-a-b{\hat{Y}}^{\prime }{}_{K}\left(n\right)\;\;\;\;\;\;\;\;\;\;\;\;\;\;\;\;\;\;\;\;\;\;\;\;\;\;\;\;\;\;\;\;$$

where *a*, *b* are the coefficients that minimize the linear fit between ${\hat{X^{\prime }}}_{K}\left(n\right)$ and ${\hat{Y^{\prime }}}_{K}\left(n\right)$ in the window of *w* samples centered on sample n. If *P*(*n*) does not indicate a high degree of correlation (*P*(*n*) > 5%), ${Z^{\prime }}_{K}\left(n\right)$ is directly calculated as:

(4) $${Z^{\prime }}_{K}\left(n\right)={\hat{X^{\prime }}}_{K}\left(n\right)-{\hat{Y}}^{\prime }{}_{K}\left(n\right)\;\;\;\;\;\;\;\;\;\;\;\;\;\;\;\;\;\;\;\;\;\;\;\;\;\;\;\;\;\;$$

**STEP 6:** Once the derivatives of the wavelet components of the corrected signal have been determined, numerical integration is applied to obtain $Z_{A}\left(n\right),Z_{D1}\left(n\right),\;Z_{D2}\left(n\right),\ldots ,Z_{Dq}\left(n\right).$

**STEP 7**: Finally, the inverse wavelet transform will be performed to obtain the corrected signal in the time domain z(t) (see Figure S1b of the Supporting Information).

### 3.2. Chemicals

Nanopure water and pure ethanol were purchased from Panreac Química SLU (Barcelona, Spain). Phosphate-buffered saline (PBS) tablets for preparing 0.01 M phosphate buffer containing 0.0027 M potassium chloride and 0.137 M sodium chloride, pH 7.4, at 25 °C were purchased from Sigma Aldrich Química, S.L.U. (Madrid, Spain). NeutrAvidin (NAv), biotinylated BSA (bBSA), and sodium dodecyl sulfate (SDS) 20% solution were purchased from Fisher Scientific S.L. (Madrid, Spain). COBAS Cleaner was purchased from Sanilabo S.L. (Valencia, Spain).

### 3.3. Intrument and Devices

#### 3.3.1. Sensors

MQCM arrays (AWSensors S. L.) comprised 24 HFF-QCM sensors integrated in a 1-inch circular AT-cut quartz wafer. The fundamental frequency of the resonators in these arrays is 50 MHz, and their surfaces are flat and polished (See Figure S2 in the Supporting Information). To clean the sensors, they were exposed to UV radiation for 10 min in a UV/ozone cleaner (BioForce Nanosciences Inc., Chicago, IL, USA), rinsed with 99% pure ethanol, rinsed with bi-distilled water, dried with ultra-pure nitrogen gas (Al Air Liquide España, S.A.) and treated with UV/ozone for 10 min again.

#### 3.3.2. Sensor Electrical Characterization

AWS X24 platform (AWSensors S. L.) was used to characterize the MQCM array response. This QCMD instrument is based on a fixed-frequency phase-shift measurement technique described elsewhere [35,36]. AWS X24 is capable of simultaneously measuring the acoustic response of up to 24 HFF-QCMD sensors with a sampling rate of three samples per second per sensor, providing both resonance frequency and dissipation data. A thermal management module is embedded into the AWS X24 system to control the temperature of the MQCM array. Temperature range can be set between 20 °C and 40 °C. A flow control module (FCUPro, AWSensors S. L.) was used to generate a stable flow through the sensor channels. AWSuite software package (AWSensors S. L.) was used to control the instrument and to register the acquired data.

### 3.4. Experimental

Protein adsorption experiments were performed over the MQCM array. For that, first, the MQCM device was mounted in a custom flow measurement cell (Jobst Technologies, Freiburg, Germany) previously cleaned with COBAS cleaning solution for 30 min, followed by repeated rinsing with HCl 0.1 M and water and dried with a stream of filtered nitrogen. Flow tubing was connected to the cell to create two separate flow regions (named *S* and *R*) with 12 sensors each (see Section S3 in the Supporting Information). Resonance frequency and dissipation of every sensor in the array was monitored in real-time during the experiment. Fluidic channels were filled with PBS at a flow rate of 20 μL/min. Baseline signals were acquired for ~5 to 10 min, followed by the sample injections only in the flow region *S* but not in region *R*, which acts as reference. The first injection consisted of neutravidin (NAv) at a high concentration (100 µg/mL). The second injection consisted of biotinylated bovine serum albumin (biotinylated BSA), at a concentration of 100 µg/mL.

## 4. Results and Discussion

We applied the algorithm presented in Section 3.1 to improve the stability and reliability of an MQCM device comprising 24 HFF-QCM sensors operating at a fundamental frequency of 50 MHz. The method provides a corrected output signal *z*(*n*), with improved stability with respect to the raw sensor signal *x*(*n*), by removing the signal fraction common to the time derivative of the DWT components of the sensor and the reference. Unlike Mecea’s approach, here, we consider the different resonator absolute responses by performing a linear projection of the reference space into the sensor space, which contributes to effectively cancelling the combined effect of all external interferences, even if the absolute resonator responses to those effects do not match perfectly (Equation (3) in step 5 of the method).

In the study, we worked with pairs of resonators (see Figure 1a). The first resonator worked as a *sensor* while we considered the second one as a *reference*. Both resonators, although very close to each other (distance < 2 mm), were located in independent flow channels in such a way that we could inject sample solely over the resonator operating as a *sensor* (see Figure S2 in the Supporting Information).

We studied the performance of the algorithm under different experimental conditions.

### 4.1. Improvement of the Frequency Stability

In a first experiment, the *sensor* and *reference* fluidic channels were filled with PBS at a flow rate of 20 μL/min and then the flow was stopped. No sample was injected in the *sensor* channel. MQCM resonance frequency was monitored for one hour under controlled temperature (23 ± 0.05 °C) conditions. In this scenario, only effects intrinsic to the sensor setup such as the mechanical stress exerted by the measurement cell, electronic noise of the characterization system, or compressional waves due to the presence of a liquid over the sensor surface, among others, affect the frequency stability.

**Figure 3:**
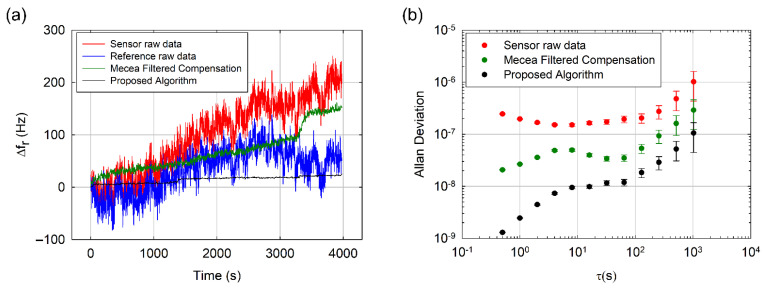
(**a**) Time evolution of the resonance frequency shifts measured at the *sensor* (red trace) and *reference* (blue trace) resonators. Corrected resonance frequency shift provided by our algorithm (black line) and by the filtered Mecea compensation method (green trace) are depicted as well. (**b**) Allan deviation vs. integration time calculated for the raw sensor resonance frequency (red dots), for the corrected resonance frequency using our algorithm (black dots) and for the corrected resonance using filtered Mecea compensation method (green dots). Error bars are included.

The monitored resonance frequency shift is shown in Figure 3a. The algorithm (black trace) greatly reduced the noise level when compared with the raw sensor signal (red trace). Sensor baseline drift also improved, from 200 Hz/h to less than 20 Hz/h. Furthermore, we compared our results with a modification of Mecea’s traditional approach [21] (green trace), which consists of applying a 16-sample length sliding averaging window to the result of the direct subtraction of the resonance frequency signals measured at the reference and at the sensor (hereinafter called the “filtered Mecea compensation method”). Averaging was applied to reduce the high-frequency noise level of the original method. Even after the additional averaging stage, our algorithm provided a better performance than the filtered Mecea compensation method both in noise rejection and drift compensation (150 Hz/h).

In order to study the stability improvement in more detail, we calculated the so-called Allan deviation (ADEV) defined in Equation (5). Allan deviation is a useful tool commonly applied to analyze the frequency stability of resonators, oscillators and clocks [20].

(5) $$\sigma_{y}\left(\tau \right)=\sqrt{\;\frac{1}{2}\langle {\left({\bar{y}}_{n+1}-{\bar{y}}_{n}\right)}^{2}\rangle }$$

In Equation (5), τ is the time between samples n + 1 and n, also called integration time, and ${\bar{y}}_{n}=\Delta f/f_{0}$ is the nth fractional frequency average over the integration time. The lower the ADEV value, the higher the resonator stability. As can be observed in Figure 3b, our algorithm offers better frequency stability for the whole τ range. Specifically, in the case of short integration times (corresponding to high frequency events in the signal), the stability enhancement is higher than two orders of magnitude when compared with the ADEV corresponding to the raw sensor signal and one order of magnitude better than the ADEV of the filtered Mecea compensation method. This improved performance relies on the high-frequency denoising capabilities of the wavelet thresholding scheme implemented (step 2 of the algorithm described in Section 3.1). When long integration times are considered (corresponding to low frequency variations in the signal), a three-fold enhancement with respect to the filtered Mecea compensation method and an improvement of up to one order of magnitude if compared with raw resonance frequency are achieved. We attribute such a good performance at reducing low frequency noise to the capability of the algorithm to suppress DWT components linearly correlated in both resonators (step 5 of the algorithm described in Section 3.1). This is achieved by performing a subtraction of the derivatives of the wavelet coefficients after a linear projection of the reference space into the sensor space, which has demonstrated to be more effective for the cancelation of this type of low frequency noise than a direct subtraction.

### 4.2. Instrument Detection Limit

**Figure 4:**
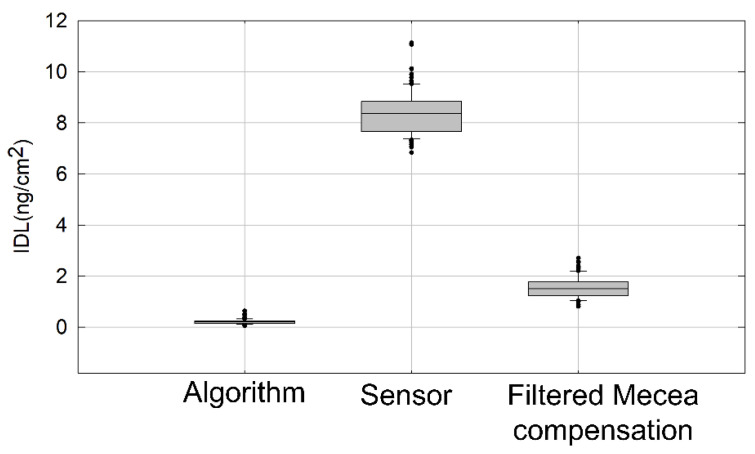
Instrument detection limit (IDL) calculated from raw sensor data, the filtered Mecea compensation method and the algorithm presented in this paper. IDL has been calculated as 3σ/S in ng/cm^2^, where σ is the system noise in Hz, and S is the sensitivity in Hz cm^2^/ng. A total of 100 intervals of the acquired baseline have been used in the calculation. Error bars are included in the figure.

The International Union of Pure and Applied Chemistry (IUPAC) states LoD as the lowest concentration of an analyte that an analytical process can reliably detect [37]. This definition considers the influence of intrinsic sensor parameters such as sensitivity, baseline stability and signal to noise ratio but also other factors such as the sensitivity and specificity of the sensor coating. Since the contribution of sensor surface functionalization to LoD depends on the application, an alternative definition is required to assess the sensor performance. Here, we use the instrument detection limit (IDL). For a gravimetric sensor, IDL is defined as the minimum surface mass that can be detected. IDL is defined as 3σ/S in ng/cm^2^, where σ is the system noise in Hz, and S is the sensitivity in Hz cm^2^/ng. QCM sensitivity is directly related to the sensor resonance frequency through the Sauerbrey equation [3]:

(6) $$S=\frac{\Delta f_{r}}{\Delta m}=-\frac{2f_{0}^{2}}{\sqrt{\eta_{q}\rho_{q}}}$$

where $\rho_{q}$ is the quartz density, $\eta_{q}$ is the AT-cut quartz shear modulus, Δm is the surface mass density in ng/cm^2^ , $f_{0}$ is the sensor fundamental resonance frequency and $\Delta f_{r}$ is the frequency shift measured at the sensor. In the case of an HFF-QCM sensor with a fundamental resonance frequency of 50 MHz, S = −5.657 Hz cm^2^/ng. As shown in Figure 4, IDL obtained by processing the raw sensor data with our algorithm is ~0.2 ng/cm^2^, which is more than one order of magnitude better than IDL obtained directly from the raw sensor data (~8.38 ng/cm^2^), and almost one order of magnitude better than the one provided by the filtered Mecea compensation method (~1.56 ng/cm^2^).

### 4.3. Removal of External Enviromental Factors

**Figure 5:**
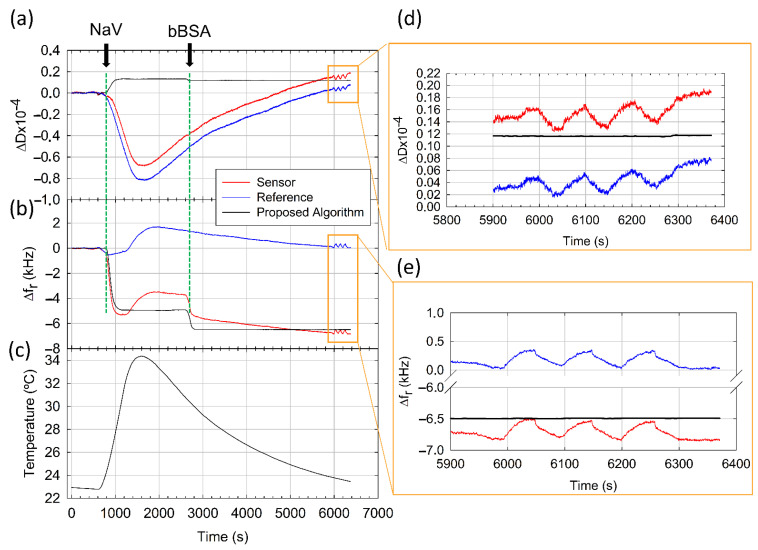
Dissipation (**a**) and resonance frequency (**b**) shifts monitored for *sensor* (red trace), *reference* (blue trace) and the algorithm result (black trace) under conditions of changing temperature and flow rate. (**c**) Temperature time evolution configured to increase from 23 °C to 34 °C and back to 23 °C. Detail of the effect of flow rate variation in dissipation (**d**) and resonance frequency (**e**) shifts.

We also tested the capability of the method to cancel the influence of unwanted external factors in resonance frequency and dissipation signals, such as temperature and flow rate gradients. PBS saline buffer was continuously flown through the *reference* and *sensor* channels (*R* and *S* regions, respectively, in Figure S2b in the Supporting Information), and only NAv and bBSA were injected sequentially in the *sensor* channel (*S* region). Both NAv and bBSA were prepared at high concentration (100 µg/mL) to ensure that adsorption reached saturation, avoiding possible differences among sensors caused by the uneven distribution of the sample in the flow measurement cell [33]. The response of one pair of resonators (*sensor*–*reference*) integrated in the MQCM device is depicted in Figure 5. To emulate a harsh environment with changing conditions, temperature control was configured to increase from 23 °C to 34 °C and to decrease back to 23 °C during the experiment (see Figure 5c). Both raw resonance frequency (Figure 5b) and dissipation (Figure 5a) shifts measured at the *reference* resonator (blue traces) were heavily distorted by the temperature effect. *Sensor* response (red traces), in turn, was affected both by the temperature shift and by the biomolecular interactions taking place on its surface.

In the case of the NAv sample injection, raw resonance frequency shift registered at the sensor is far from the typical smooth monotonically decreasing curve expected. The raw dissipation shift also shows strange behavior. If we consider that the NAv adsorption process reaches a plateau at 2500 s, the frequency shift is −3681 Hz, corresponding to a mass density of 650 ng/cm^2^, and a dissipation shift of −43 × 10^−6^. These results do not match well with the literature, where typical values reported for mass density are greater than 750 ng/cm^2^ and negative Δ*D* values have not been described [29,30,31,32]. These results do not match either with control experiments carried out at 23 °C (see Table 1).

Regarding bBSA injection, although baseline stabilization is not complete because of the temperature influence, we considered that a plateau was reached at 5500 s, where raw Δ*f*_r_ is −2818 Hz (498 ng/cm^2^) and Δ*D* is 52 × 10^−6^. Theoretically, bBSA should be adsorbed over NAv as a monolayer, giving an approximate mass value of 250 ng/cm^2^ [33]. Furthermore, there is a significant mismatch with control experiments shown in Table 1.

At this point, we applied our algorithm to disentangle contributions arising from temperature and from protein adsorption. Corrected resonance frequency and dissipation shifts are depicted as black traces in Figure 5a,b, respectively. Both corrected signals show a typical behavior characteristic of a protein adsorption experiment carried out under constant temperature conditions (see Figure S3 in the Supporting Information). After the correction, NAv and bBSA resonance frequency and dissipation shifts are in good agreement with the literature data [29,30,31,32] and control experiments. We conjecture that small deviations between control and validation experiments showed in Table 1 are related to sample preparation and to the temperature shift effect on the protein adsorption process.

Finally, we evaluated the capability of the method to correct additional flow rate gradients. To investigate this point, we modified the flow rate between 15 and 35 µL/min from 6000 to 6300 s (see Figure 5). This periodic variation resulted in a sawtooth-shaped interference that affects both resonators. Since the *sensor* and *reference* are located in different flow channels, interference is highly correlated but not identical in both resonators. Nevertheless, the proposed method adequately removed the effects of the flow rate variation both in resonance frequency and dissipation shifts, as is shown on insets (e) and (d) in Figure 5, respectively. We consider it important to underscore that temperature was not yet stable at 6000 s (see Figure 5c). Thus, our method effectively cancelled the combined effect of both factors, e.g., temperature and flow rate variation.

## 5. Conclusions

We presented a method that exploits the high level of correlation found in the response of the acoustic wave sensors integrated in an MQCM device to minimize the impact of unwanted disturbances on the stability and reliability of the measurement. The method is based on the hypothesis that interferences caused by harsh environmental conditions (such as temperature or pressure) or factors intrinsic to the sensor setup (such as electronic noise or stress caused by the measurement cell) can be effectively removed from resonance frequency and dissipation by combining DWT with an algorithm to cancel signal components common to two resonators, i.e., sensor and reference. The method is robust against variations in the absolute sensor responses to those external or internal factors.

When tested under static flow conditions and controlled temperature (23 ± 0.05 °C) the method showed an IDL enhancement of almost two orders of magnitude with respect to the original sensor data. Furthermore, the method successfully minimized the effect of changing temperature (between 23 °C and 34 °C) and flow rate (between 15 and 35 µL/min) in protein adsorption experiments.

To the best of the authors’ knowledge, no other method has been previously proposed in the literature to correct both dissipation and resonance frequency against external and internal disturbances in QCMD. In the near future, we plan to apply these results to develop a portable immunosensor system for on-site monitoring of pesticides and antibiotics in honey samples.

## Figures and Tables

**Table 1:** Comparison of resonance frequency and dissipation shifts measured during a control experiment (see S4 in the Supporting Information) and the results provided by the algorithm in the experiment showed in Figure 5 under strong influence of external factors. Control experiment was carried out under stable temperature (23 °C) and flow rate (20 µL/min). Data shown in the table represent average and standard deviation calculated from 4 HFF-QCMD sensors integrated in a column of the MQCM device.

	NAv		bBSA	
	Control Experiment	Algorithm Result	Control Experiment	Algorithm Result
Δf_r_ (Hz)	−5454 ± 193	−5002 ± 125	−1335 ± 121	−1558 ± 63
ΔD (10^−6^)	13.7 ± 3.5	16 ± 6.5	1.8 ± 3.6	−0.8 ± 0.2

## Data Availability

Not applicable.

## Supplementary Materials

The following are available online at https://www.mdpi.com/article/10.3390/s21124166/s1: wavelet Daubechies transform, energy distribution of DWT coefficients, experimental setup, control experiments.
