# Supplementary material for: A Real-Time Method for Improving Stability of Monolithic Quartz Crystal Microbalance Operating under Harsh Environmental Conditions
Source: Sensors (Basel). 2021 Jun 17;21(12):4166. doi: 10.3390/s21124166 (PMC8235264; doi:10.3390/s21124166)
Supplement: Supplementary file 1 [file sensors-21-04166-s001.zip › sensors-1233671-supplementary.pdf]

sensors

Article

A Real-Time Method for Improving Stability of Monolithic Quartz Crystal Microbalance Operating under Harsh Environmental Conditions. Supporting Information

Román Fernández [1,2,*], María Calero [2], Yolanda Jiménez [2] and Antonio Arnau [2]

[1] Advanced Wave Sensors S.L. Paterna, 46988 Valencia, Spain
[2] Centro de Investigación e Innovación en Bioingeniería, Universitat Politècnica de València, 46022 Valencia, Spain; macaal3@teleco.upv.es (M.C.); yojiji@eln.upv.es (Y.J.); aarnau@eln.upv.es (A.A.)
* Correspondence: rfernandez@awsensors.com

S1. Wavelet Daubechies transform

Direct and inverse wavelet transform is applied in the method presented. Concretely, a Daubechies wavelet transform with three vanishing moments (db3) and 4 decomposition levels is used [1]. Figure S1 shows the Mallat scheme used for signal decomposition and reconstruction. The decimation process previous to each stage reduces the number of samples in the subsequent stage by a factor of 2.

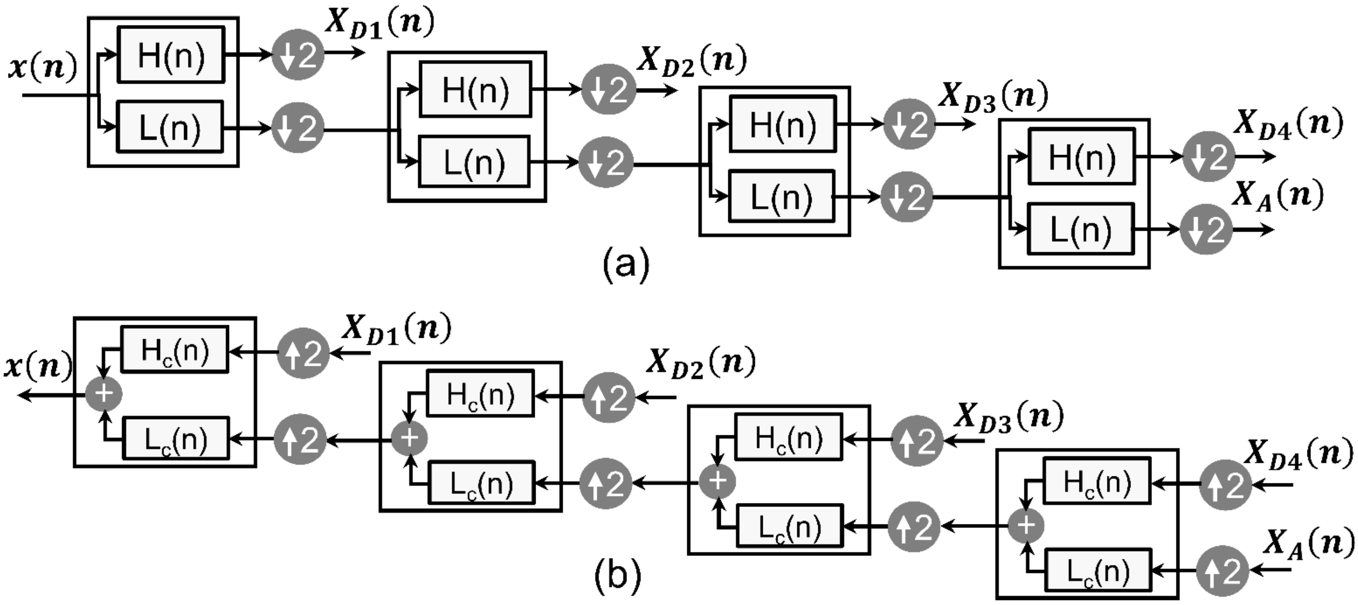

Figure S1. Mallat tree decomposition (a) and reconstruction (b) schemes implemented. H(n) and L(n) represent the high and low frequency decomposition filters respectively. H_c(n) and Lc(n) represent the high and low frequency reconstruction filters respectively. $X_A(n)$ refers to the approximation coefficient expansion and $X_{Dq}(n)$ represent the detail coefficient expansions of the different decomposition levels q. ↑ 2 symbol represents an interpolation operation by a factor of 2 while ↓ 2 represents a decimation by a factor of 2.

S2. Energy distribution of DWT coefficients

Table S1 shows the energy contribution of each DWT coefficient to the total wavelet transform energy in a typical protein adsorption experiment. Energy is clearly concentrated in the approximation coefficient $X_A(n)$, since the main components of its spectrum are located in low frequency subband.

Table S1. Energy distribution of the different DWT coefficients of Δf_r and ΔD monitored in a typical protein adsorption experiment.

	$\boldsymbol{X_A(n)}$	$\boldsymbol{X_{D1}(n)}$	$\boldsymbol{X_{D2}(n)}$	$\boldsymbol{X_{D3}(n)}$	$\boldsymbol{X_{D4}(n)}$
Δf_r (%)	99.9998	$9.2 \cdot 10^{-5}$	$6.1 \cdot 10^{-5}$	$4.6 \cdot 10^{-5}$	$4.46 \cdot 10^{-5}$
ΔD (%)	99.9947	0.0022	0.0014	0.0009	0.0008

S3. Experimental Setup

This section details the Monolithic QCM setup used in the real time monitoring of the protein adsorption experiment. Figure S2 (a) and (b) show top and bottom surfaces of the MQCM device, respectively. Array consists of 6 columns of 4 HFF-QCM resonator elements each. Array elements are based on a 50 MHz one-sided inverted MESA geometry and are optimized in terms of size and electrode geometry to facilitate manufacturing and integration with fluidics and electronics, spurious mode suppression, and operation in liquids. The top electrode is grounded, and it is common to all 24 array elements. This common electrode constitutes the "working" side of the array. It faces the sample. On the other side of the array, the short rectangular electrodes are connected to the driving circuitry individually.

Figure S2 (c) shows the array mounted in the flow measurement cell. Measurement cell is divided into 6 independent flow channels covering 4 sensors each. Each channel has an inlet and an outlet that can be connected to flow tubing through steel cannulas. Flow connections of the array sensor measurement cell were configured to create two separate flow regions with 12 sensors each. Region *R* contains the *reference* resonators while Region *S* contains the resonators operating as *sensors*. Figure S2 (b) and (c) show a schematic representation of both regions, *R* region columns are imbricated with *S* region columns to achieve a configuration in which each *sensor* has a *reference* one aside.

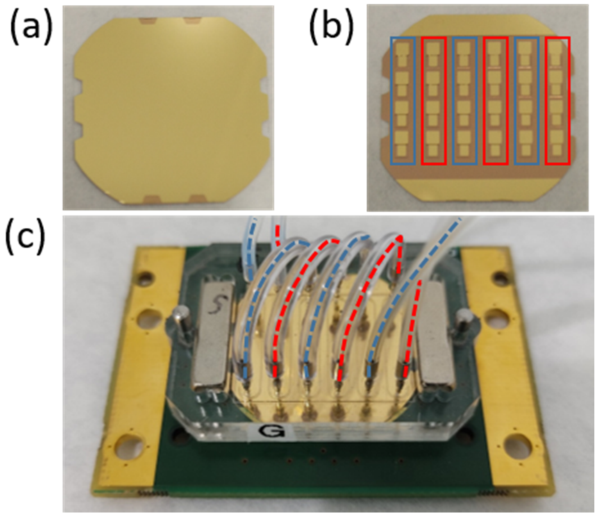

Figure S2. (a) Top and (b) bottom surfaces of the Monolithic QCM device. (c) MQCM device mounted in the flow cell. A blue dotted line is used to mark the tubing of region *R* that comprises 12 *reference* resonators while a red dotted line is used to mark the tubing of region *S* that comprises 12 resonators operating as *sensors*.

S4. Control Experiments

A set of control experiments were carried to determine the response of the NAv and bBSA protein under controlled conditions. Instrument temperature was set to 23 °C and flow rate was configured to be 20 µL/min. Figure S3 shows monitored resonance frequency and dissipation shifts at 4 different sensors integrated in the same channel of a MQCM device.

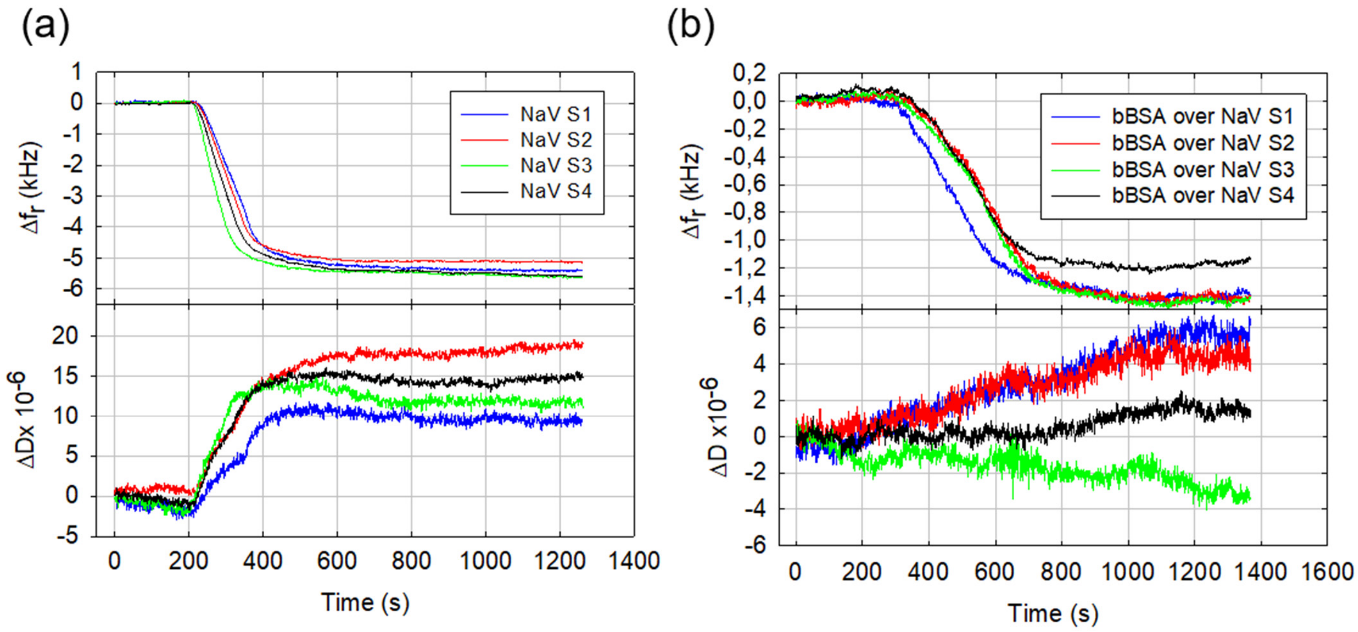

Figure S3. (a) Resonance frequency (top) and dissipation (bottom) shifts acquired in 4 HFF-QCMD sensors integrated in a MQCM channel during a NAv over gold adsorption experiment. (b) Resonance frequency (top) and dissipation (bottom) shifts acquired in 4 HFF-QCMD sensors integrated in a MQCM channel during a bBSA over NAv adsorption experiment.

References

1. Mallat, S. *A Wavelet Tour of Signal Processing*; 2008;
